# Tricuspid Endocarditis: A Case Report and Comprehensive Literature Review

**DOI:** 10.7759/cureus.24027

**Published:** 2022-04-11

**Authors:** Sherif Elkattawy, Ramez Alyacoub, Iman El-Feki, Hardik Fichadiya, Edmund Appiah-Kubi, Jesus Romero, Xutong Guo, William Edward

**Affiliations:** 1 Internal Medicine, Rutgers-New Jersey Medical School/Trinitas Regional Medical Center, Elizabeth, USA; 2 Internal Medicine, St George's University, West Indies, GRD; 3 Internal Medicine, Trinitas Regional Medical Center, Elizabeth, USA; 4 Cardiology, Trinitas Regional Medical Center, Elizabeth, USA

**Keywords:** infective endocarditis, tricuspid, endocarditis, vegitations, valve

## Abstract

Infective endocarditis is a multisystem disease. Tricuspid valve endocarditis is frequently seen in patients with intravenous (IV) drug users. Cavitating lung nodules predominantly in a peripheral location in IV drug users indicate the possibility of septic emboli. Large vegetation and persistent bacteremia with septic embolic phenomena are the most common indication for surgery.

We present a case of a 62-year-old male with a history of IV drug use who presented with epigastric abdominal pain, pleuritic chest pain, and shortness of breath. CT chest showed cavitating lung nodules suggestive of septic pulmonary emboli. A transesophageal echocardiogram (TEE) showed tricuspid valve vegetation despite a normal transthoracic echocardiogram. The patient was treated with intravenous antibiotics. He was deemed a poor surgical candidate; therefore, he was transferred to a tertiary center for AngioVAC (AngioDynamics, Latham, New York).

## Introduction

Infective endocarditis is a disease affecting the endocardial surface of the heart, usually involving the heart valves [1]. This disease occurs when a bacterial or fungal pathogen enters the blood through intravenous catheter use, dental procedures, or surgical procedures and attaches to the inner lining of the heart [2]. The leading bacterial cause of infective endocarditis (IE) is Staphylococcus aureus, followed by Streptococcus viridans with rare presentations by Staphylococcus epidermidis, Streptococcus bovis, and HACEK organisms (Haemophilus species, Aggregatibacter species, Cardiobacterium hominis, Eikenella corrodens, and Kingella species). As of 2011, the incidence of endocarditis has risen to 15 cases per 100000 persons per annum, with older male populations presenting with a higher risk of infection [1]. Infective endocarditis affects multiple organ systems and presents with clinical features such as fever, fatigue, murmurs, anemia, emboli, and, lastly, painful lesions on fingers or toes [2]. Diagnosis of IE is made by Duke criteria, which include clinical criteria, blood cultures, and imaging with echocardiography. Transthoracic echocardiography or transesophageal, which is more sensitive, detects tiny vegetations (1 mm to 1.5 mm) on valve leaflets [2]. Management involves implementing a long course of antibiotics, usually for four to six weeks, to eradicate the causal organism [1].

## Case presentation

A 62-year-old African American male with a past medical history of hepatitis C, IV heroin abuse, and cocaine use disorder presented to the ED, with the chief complaint of acute, sharp, constant left upper quadrant abdominal pain for the past three days. Symptoms were noted to be associated with pleuritic chest pain, shortness of breath, and nausea. On review of systems, the patient denied chills, fever, edema, congestion, cough, constipation, vomiting, altered mental status, dizziness, and headache. Social history is significant for cigarette, heroin, and cocaine use for the past 20 years. The patient reported smoking six cigarettes a day and using heroin intravenously or intranasally. The last heroin use was six days ago via IV. The patient denies alcohol usage. His family history was insignificant.

Vitals on admission were stable. The patient was afebrile, pulse rate was 89 bpm, respiratory rate was 18 breaths per min, O_2_ saturation was 99% on room air, and blood pressure was 112/72. Physical exam was significant for a moderately tender abdomen in the left upper quadrant and epigastric region. Heart rate was regular, with no murmurs or extra heart sounds appreciated on auscultation. Lungs were clear bilaterally with vesicular breath sounds.

**Figure 1:**
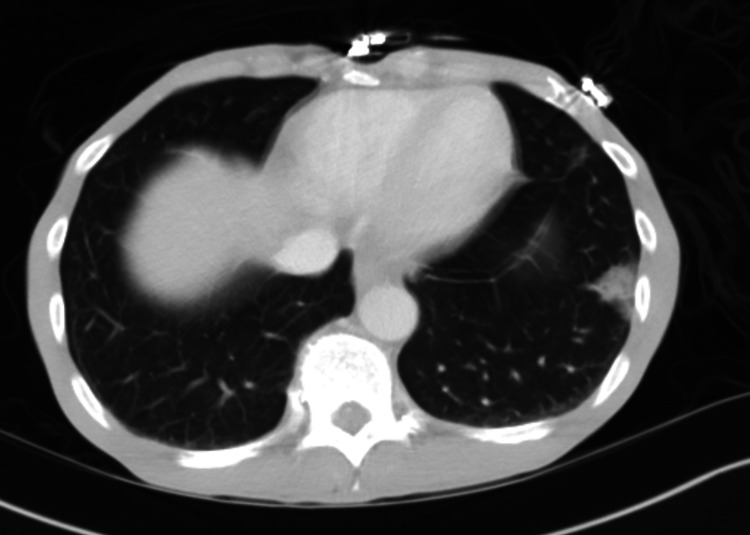
CT abdomen shows a peripheral ground-glass opacity involving the left lower lobe of the lung

CT of the abdomen showed a peripheral ground-glass opacity involving the left lower lobe of the lung (Figure 1).

**Figure 2:**
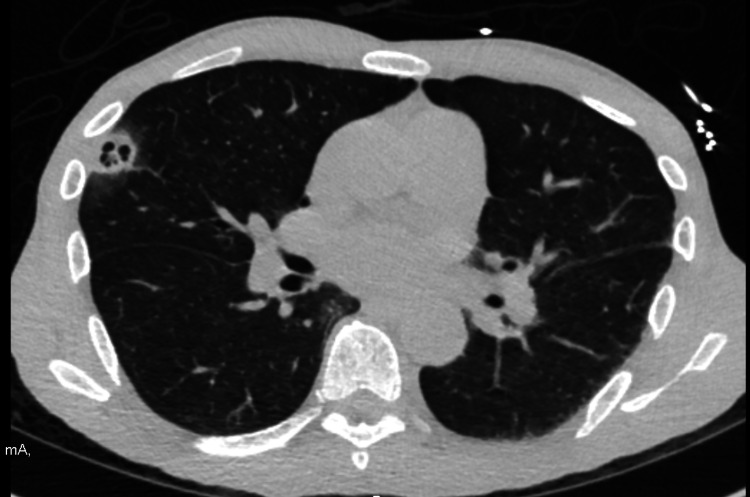
CT chest shows peripheral cavitary opacities in the right upper lobe

CT of the chest showed an additional multiseptate cavitary lesion in the right upper lobe (Figure 2), precocious for septic embolism. Urine culture was positive for cocaine. WBC was increased to 16. Blood culture showed Methicillin sensitive Staphylococcal aureus. HIV and rapid plasma reagin (RPR) were negative.

**Figure 3:**
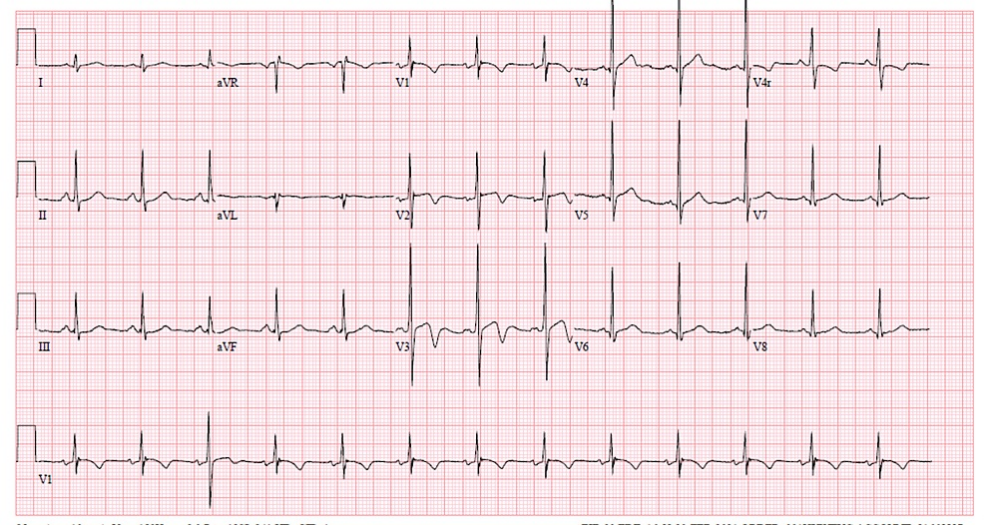
EKG shows sinus rhythm and nonspecific T wave abnormalities

 ECG showed normal sinus rhythm with right ventricular hypertrophy with strain pattern (Figure 3).

Transthoracic echocardiogram showed left ventricular ejection fraction (LVEF) of 65-70% with no abnormalities noted in all four chambers and no vegetation visualized on leaflets. It also showed a normal tricuspid valve structure with a regurgitation velocity of 2.45 m/s and a 3 cm pedunculated mobile vegetation on the anterior leaflet of the bicuspid valve. See Video 1.

**Video 1:** Transesophageal echo showing tricuspid vegetation

Based on Duke criteria, infective endocarditis diagnosis was made based on positive blood bacterial cultures and transesophageal echocardiogram (TEE) vegetation findings. According to guideline-directed medical therapy for tricuspid bacterial endocarditis, the patient was treated with high-dose IV nafcillin, aspirin, atorvastatin, famotidine, ferrous sulfate, and oxycodone-acetaminophen. Cardiology and cardiothoracic surgery were consulted. Due to his extensive history of IV drug abuse and poor medication compliance, the patient was deemed a poor candidate for valve replacement. However, this patient with large vegetations and pulmonary septic emboli continued to have recurrences of fever and rising leukocytosis. He is a candidate for vegetation extraction by AngioVac (AngioDynamics, Latham, New York) after evaluation by cardiothoracic surgery. He was then transferred to a university hospital for AngioVAC and continued care.

## Discussion

Of all the causes that predispose to right-sided valve endocarditis, IV drug use (IVDU) is the most common culprit in patients who present with tricuspid valve endocarditis (TVIE). It accounts for 30-40% of all cases [3-4]. Other causes include indwelling lines and cardiac implantable devices. In order to establish a diagnosis of definite endocarditis, the modified Duke criteria have been widely used by clinicians and demonstrate that the patient must meet the following: two major criteria, one major with three minor criteria, or five minor criteria [4]. However, it is important to note that these criteria have lower sensitivity in evaluating patients with prosthetic valve endocarditis or cardiac device infection [5]. The diagnosis of infective endocarditis is initiated with a TTE [6]. However, studies had shown that TTE is limited in successfully detecting valvular vegetations compared with a TEE, which was evident in our patient’s case when initial TTE failed to identify a 3 cm vegetation on the tricuspid valve.

Management depends on several factors such as the size of vegetation and the severity of the disease. Nonetheless, most cases of TVIE are treated initially with empiric antibiotics, and studies have shown that antibiotics alone eliminate the bacteremia in 70-85% of the cases. Adjusting the antibiotic regimen after receiving culture sensitivity results has proven to have better outcomes [7], and the most common organism to cause TVIE is Staphylococcus aureus. Treatment includes oxacillin against methicillin-sensitive Staphylococcus aureus (MSSA), daptomycin, and vancomycin plus gentamicin for Staphylococcus aureus bacteremia ceftriaxone for Streptococci and ceftriaxone plus ampicillin for Enterococci organisms [7]. Blood cultures were positive for MSSA in our patient, and thus, high-dose IV nafcillin was started for optimal treatment.

On the other hand, surgical intervention is reserved for patients with any of the following criteria: TV vegetation >2 cm with septic pulmonary emboli, persistent bacteremia for one week despite adequate treatment, or severe tricuspid regurgitation with right-sided heart failure [8].

Our patient fit one of the described criteria above, presenting with tricuspid valve vegetation of 3 cm with septic pulmonary emboli as evidenced on chest CT scan. Due to his extensive history of drug abuse, the patient was a poor candidate for valve replacement surgery. Although the mortality rate after surgery is lower in IVDUs than non-IVDUs, the rate of valve re-infection and reoperation is notably higher [7]. Given that our patient was a poor surgical candidate, the presence of large vegetation (3 cm), septic pulmonary emboli, and continued fever despite appropriate antibiotics, transcatheter aspiration via the AngioVac system was justified [6]. The AngioVac has the benefit of vegetation debulking, therapy decreasing the bacterial load, and hastening antibiotic clearance of septicemia [9].

## Conclusions

In conclusion, tricuspid valve endocarditis is common in patients with intravenous drug use. It can cause septic pulmonary emboli presenting as cavitating lung nodules. Large vegetation and persistent bacteremia with septic embolic phenomena are the most common indications for surgery.
